# Illuminating pathogen–host intimacy through optogenetics

**DOI:** 10.1371/journal.ppat.1007046

**Published:** 2018-07-12

**Authors:** Ruben Dario Arroyo-Olarte, Laura Thurow, Vera Kozjak-Pavlovic, Nishith Gupta

**Affiliations:** 1 Department of Molecular Parasitology, Faculty of Life Sciences, Humboldt University, Berlin, Germany; 2 Department of Microbiology, Biocenter, Julius Maximilian University, Würzburg, Germany; University of Wisconsin Medical School, UNITED STATES

## Abstract

The birth and subsequent evolution of optogenetics has resulted in an unprecedented advancement in our understanding of the brain. Its outstanding success does usher wider applications; however, the tool remains still largely relegated to neuroscience. Here, we introduce selected aspects of optogenetics with potential applications in infection biology that will not only answer long-standing questions about intracellular pathogens (parasites, bacteria, viruses) but also broaden the dimension of current research in entwined models. In this essay, we illustrate how a judicious integration of optogenetics with routine methods can illuminate the host–pathogen interactions in a way that has not been feasible otherwise.

## Shine on me: Twinkling the murky microbial world

Infectious diseases are a product of the complex interplay between a particular host and pathogen. In the case of intracellular pathogens, another layer of intricacy is added. The use of chemicals to control or monitor cellular pathways within pathogens or within sheltering host cells has an inadvertent effect on the other entity, which often complicates the data interpretation. Optogenetics holds the promise to resolve this enduring concern by expressing light-sensitive proteins to examine a pathway of interest in intertwined models. The method combines accuracy, efficiency, and speed with extremely high spatial and temporal resolution in altering or measuring the activity of a target pathway [1,2]. It offers other advantages as well when controlling cellular signals for studying infection processes: (a) a reversible and conditional switching; (b) circumvention of difficulties often faced with chemicals, i.e., poor diffusion, premature degradation, constant activation, and pleiotropic effects; (c) gene-encoded expression, thus inheritable to the progeny and uniform response in a clonal population. Not everything about this method is shining, though. Just as with any technology, there are credible issues varying from model to model. These include the general toxicity of light-responsive proteins, basal (dark) activity in regular cultures, and sensitivity of some organisms to a particular spectrum or intensity of light. The making of optogenetically modified cells (via plasmid transfection or viral vectors) expressing certain tools may cause a perturbation in cell behavior, but it can be minimized by standard optimization (i.e., promoter strength, tool screening, dark culture, inclusion of proper controls) and/or by using inducible expression systems. The advantages of optogenetics so far have far outweighed the stated concerns, as evident by its exceptional success.

Originally, optogenetics was comprised of light-activated proteins that can modify membrane potential or allow control of signal cascades, molecular interactions, and gene expression [2,3]. The ever-expanding field now, in its broadest sense, includes gene-encoded light-sensitive sensors, which can be deployed to gauge intracellular messengers or metabolites. Not least, the method has also embraced technological procedures to deliver light-regulated proteins, to control the illumination, and to measure the outcome [1–3].

Currently, more than 40 types of optogenetic actuators and around 30 biosensors are available according to Addgene repository (www.addgene.org). It is worth noting that many of them have been catered to address a wide range of hypotheses in neurobiology. While other research fields have begun adopting them to meet specific objectives, such as to study the stage differentiation in intracellular parasites [4] or to fine-tune the function of immune cells [5], applications of light-activated proteins remain extremely limited. We believe that the technique is now well primed to answer prevailing questions and shepherd new avenues in infection research.

**Fig 1:**
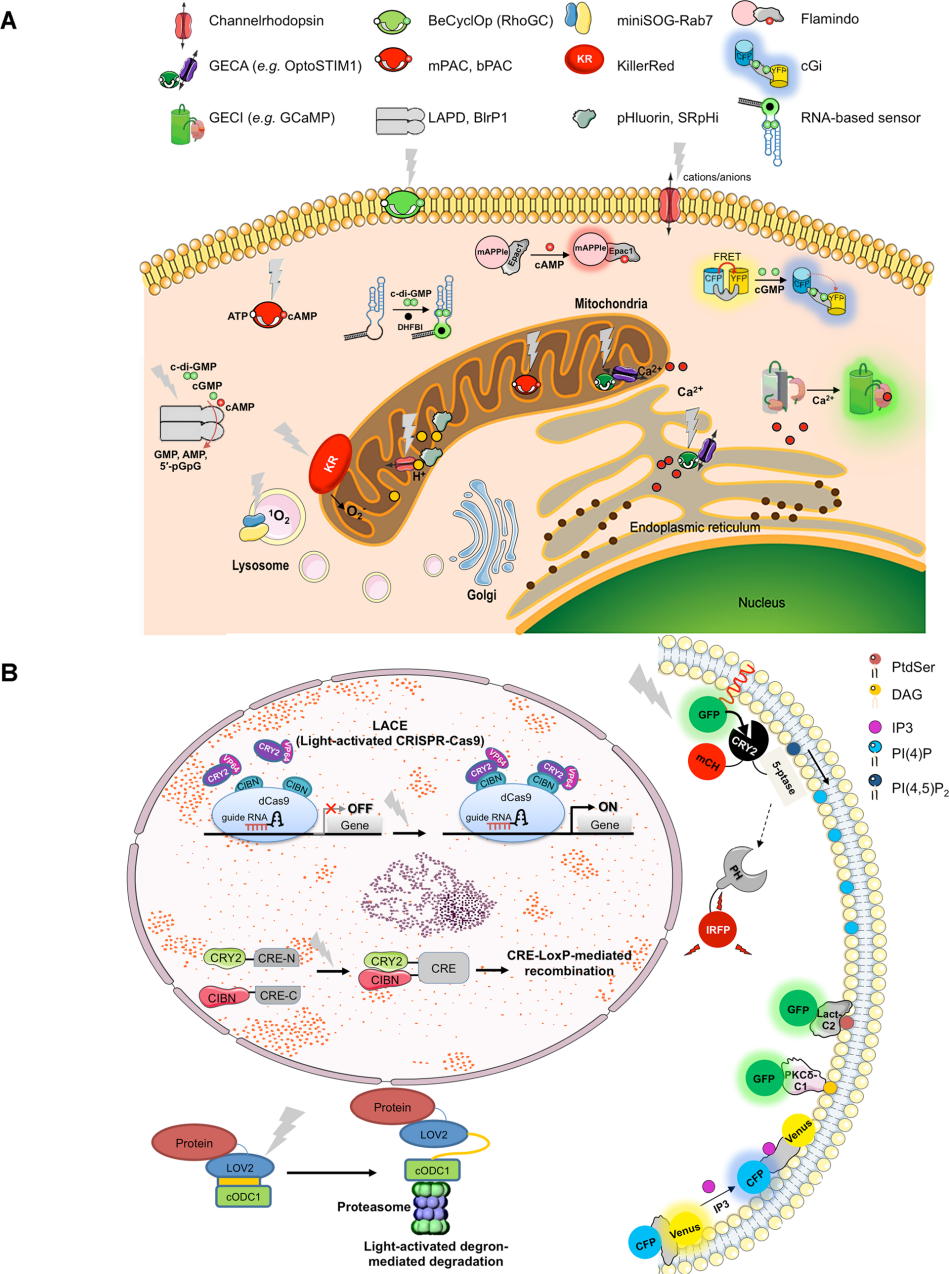
Optogenetic tools proposed for infection biology. (A) Scheme of selected opto-tools to modulate and/or monitor second messengers, ions, and pH. Indicated proteins can be targeted to the prokaryotic/eukaryotic pathogens or even to the organelle of choice in the host cell by means of corresponding sorting signal sequence. For simplicity, only few selected organelles of a typical eukaryotic cell are shown to illustrate the concept. The actual proteins for individual applications may differ in their domain structure, mode of action, and light absorption. (B) Light-regulated methods to control gene expression, protein stability, and phosphoinositide signaling, as well as biosensors of lipids and lipid-derived metabolites. Upon illumination, an RNA-guided dCas9 binds to a CRY2-VP64 transactivation domain, which in turn allows otherwise repressed transcription of a gene. LoxP-mediated recombination at a target locus is achieved by a photo-dimerizable CRE recombinase. Light-activated degron: The protein of interest is fused to a photosensitive LOV2 and a proteasome targeting cODC1 domain. Optically induced degradation is facilitated by a conformational shift in the latter 2 domains. CRY2/CIBN fusion to inositol phosphatase enables a concurrent modulation and evaluation of phosphoinositide metabolism. Lipid-binding domains Lact-C2 and PKCδ-C1 fused to GFP allow fluorescent detection of subcellular PtdSer and DAG, respectively. Equally, a fusion of CFP and Venus with IP_3_-binding motif permits a FRET-based monitoring of IP_3_. Further details on indicated proteins can be found in S1 Appendix and references therein. CFP, cyan-fluorescent protein; CIBN, N-terminus of CIB1; CRE, cyclization recombinase; DAG, diacylglycerol; FRET, fluorescence-resonance energy transfer; GFP, green fluorescent protein; LACE, light-activated CRISPR-Cas9 effector.

**Fig 2:**
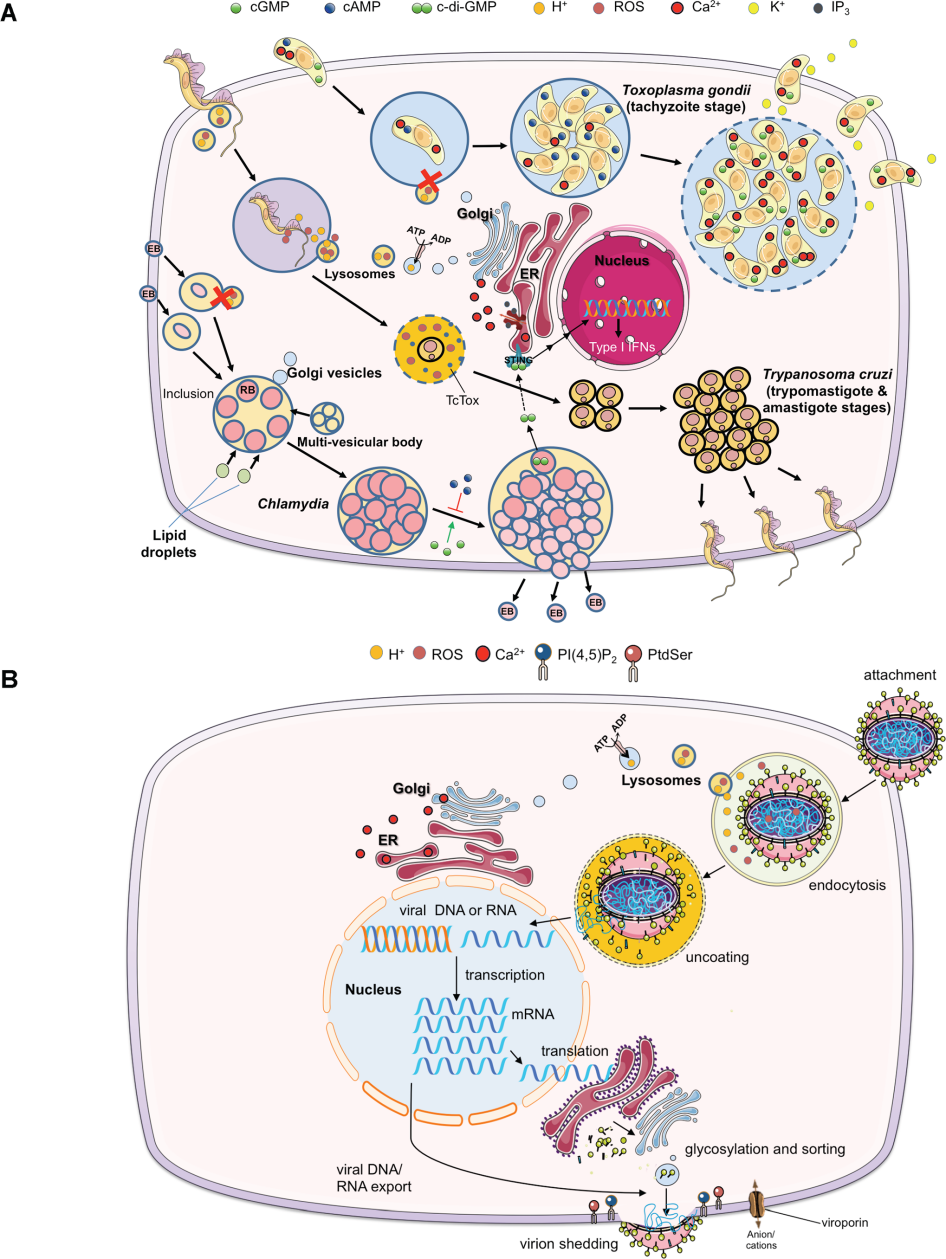
Asexual reproduction of prototypical intracellular pathogens within a mammalian host cell. (A) Strategic stages during the development of 2 parasitic protists (*Toxoplasma gondii*, *Trypanosoma cruzi*) and a bacterium (*Chlamydia*). Note that only selected features are highlighted. The shared events include invasion, proliferation, and egress. The tachyzoite stage of *T*. *gondii* actively invades host cells, reorders several organelles (not depicted for simplicity), replicates by endodyogeny in a nonfusogenic vacuole, and then exits by lysing the vacuolar and host membranes. Cyclic nucleotides (cAMP, cGMP) and ions (Ca^2+^, K^+^) play very important roles during the lytic cycle. The trypomastigote stage of *T*. *cruzi* enters the host cell by recruiting lysosomes and then escapes into cytoplasm (mediated by *Tc*Tox), where they reproduce asexually as amastigotes. Among others, Ca^2+^, pH, and ROS are major factors during *T*. *cruzi* infection. The EBs of *Chlamydia* are endocytosed into membranous vacuoles, which fuse to form an inclusion, the replicative compartment. Later on, they differentiate into larger metabolically active RBs, which replicate by binary fission before converting back to EBs. Similar to tachyzoites, *Chlamydia* is known to intercept/recruit many host organelles, such as Golgi, lipid droplets, and endolysosomes, probably for acquiring nutrients. Again, cAMP and cGMP, along with prokaryote-specific c-di-GMP, control the stage differentiation and STING-mediated modulation of host immunity genes, respectively. (B) Abridged lifecycle of viruses infecting a host cell. Key second messengers, ions, and metabolites potentially regulatable or detectable by optogenetic means are shown in relation to specific events during the course of infection. In particular, calcium, pH, ROS, and phosphoinositide signaling regulate a repertoire of phenomena. For additional details, please refer to the table outlining different tools, pathogens, and paradigms (Table 1). EBs, elementary bodies; RBs, reticulate bodies; ROS, reactive oxygen species; STING, stimulator of interferon genes; *Tc*Tox, *T*. *cruzi* toxin (hemolysin).

Herein, we outline comprehensive applications of optogenetics to study various paradigms embracing intracellular parasites, bacteria, and viruses of clinical as well as veterinary relevance (Table 1 and Fig 1). Specifically, Fig 1A shows selected opto-tools to modulate or sense secondary messengers (cyclic nucleotides) and physicochemical parameters (pH, reactive oxygen species [ROS], ions), whereas Fig 1B highlights the light-induced control of gene editing, protein expression, and lipidic signaling. A list of relevant references including Addgene construct numbers is included as supporting information (S1 Appendix). The text below explains how best we can deploy them to examine notable events during asexual reproduction of archetypical pathogens (Fig 2). Although we outline only designated pairs of optogenetic proteins and pathogens, in principle, most systems are equally applicable to all genetically tractable organisms. In fact, with the advent of CRISPR-Cas9 (clustered regularly interspaced short palindromic repeats-associated protein 9) and related genome engineering tools in common parasitic protists [6] and other microorganisms [7], it has become easier than ever to make desired optogenetic strains in “model” as well as “nonmodel” pathogens.

**Table 1:** Exemplary optogenetic tools to study specific paradigms of the pathogen–host interactions.

Protein family	Optogenetic tools	Parasite or host cell	Bacteria or host cell	Virus-infected host cell
		(potential usage)	(potential usage)	(potential usage)
**Opsin**	Channelrhodopsin (K^+^, Na^+^, Ca^2+^, H^+^, Cl^-^)	Reversible perturbation of ion homeostasis in parasite and host cells; Organelle-specific ion uncoupling	Study the relevance of K^+^ homeostasis against toxic electrophilic compounds in gram-negative bacteria; Modulation of mitochondrial function to study the pathogen’s dependence on the organelle (e.g., *Chlamydia*, *Salmonella* etc.)	Study of subcellular ion pools in host organelles; Functions of viral membrane channels (viroporins) during lifecycle
**GECA, GECI**	PACR, light-activated GPCRs/RTKs, OptoSTIM1, GCaMP_1-6_, YC-Nano	Manipulation of calcium signaling; Examining of Ca^2+^ dynamics/flux between organelles during lytic cycle	Oscillation and detection of calcium levels (e.g., *Chlamydia* & *Mycobacteria*)	Conditional manipulation of virus-induced perturbation of host-cell Ca^2+^ to promote viral replication and inhibit immune response
**Cyclic nucleotide cyclase and sensor**	bPAC and mPAC for cAMP, BeCyclOp (RhoGC) and bPGC (BlgC) for cGMP, BphS for c-di-GMP, Flamindo_1-3_ (cAMP), cGi and FlincG_1-3_ (cGMP), RNA-based sensor (c-di-GMP)	Induction and concurrent monitoring of cNMP signaling during lytic cycle and stage differentiation of parasites	Modulation of transcription and mitochondrial function during *Chlamydia* and *Salmonella* infection; Control of cAMP-mediated autophagy in cells infected with *Staphylococcus* and *Mycobacteria*; Induction of c-di-GMP during *Staphylococcus* infection and control of biofilm formation and vector colonization, as seen in *Borrelia*	Alteration of cAMP-dependent resistance to *Vibirio cholera* bacteriophages; Stimulation of cGMP-AMP–dependent antiretroviral response (HIV-1)
**Phosphodiesterase**	LAPD (cAMP, cGMP), BlrP1 and EB1 (c-di-GMP)	Knockdown of cNMP signaling during lytic cycle and stage differentiation (*Toxoplasma*, *Plasmodium*); Adaptation to hypoxia (*Leishmania*); Regulation of virulence factors, cell death, and osmoregulation (*Trypanosoma*)	Repression of cyclic nucleotide signaling in conjunction with activation by nucleotide cyclase (see above)	Modulation of virus production and pathogen phagocytosis by infected cells through cAMP/ cGMP (HIV-1, Measles virus)
**Genome editing, transcription, protein stability, and epigenetics**	LACE, LOV2-ODC/B-LID degron, LITE	Activation or repression of proteins in parasite or host cell; Alteration of epigenetic states	Bi-directional control of gene expression in bacteria or host cell	Regulation of immunity-related genes & gene editing of viral proteins
**ROS-generating proteins**	KillerRed (superoxide), miniSOGs (singlet oxygen)	Regulation of ROS-mediated killing and host oxidative stress on *Trypanosoma cruzi*	Induction and detection of ROS during infection, e.g., *Chlamydia*	Role of ROS induction (plant virus, HIV-1)
**Lipid actuators**	CRY2/CIBN fusion coupled to inositol phosphatase	Phosphoinositide signaling during replication and stage differentiation	Alteration of host phosphoinositide levels during infection (e.g., induction of phagocytosis by *Yersinia* and *Listeria*)	Control of viral attachment and fusion to host-cell plasma membrane; Lipid rafts as platform for viral particle assembly
**Protein recruiters and oligomerizers**	(1) CRY2/CIBN fusion to Cre system (2) CRY2/CIBN fusion to GTPase (3) CRY2 fusion to antiviral oligomers (4) PhyB/PIF coupled to Tiam-DH-PH domain	Light-activated Cre-mediated recombination to delete virulence factors (*Leishmania*, *T*. *cruzi*); Changes in cytoskeleton and repositioning of host-cell organelles (*Toxoplasma*, *Plasmodium*)	Control of Rho/Ras GTPase signaling to alter host actin-cytoskeleton polymerization (e.g., *Clostridium*, *Vibrio*, *Bordetella*)	(1) Deletion of integrated proviral DNA (HIV-1) (2) Perturbation of replication and assembly of viral particles (dengue) (3) Study of oligomerization and subcellular redistribution of the interferon-inducible IFI16 upon herpesviridae infection (4) Subversion of the actin cytoskeleton to promote viral entry, trafficking and spread
**Gene-encoded metabolite sensors**	Lact-C2-GFP (PtdSer), PKCδ-C1 and PKD-C1 (diacylglycerol), PASS (PtdOH), small soluble metabolite sensors (sugars, amino acids, lactate *etc*.)	Monitoring of lipid trafficking, drug inhibition and metabolic transport in parasitized cells	Monitoring of lipid trafficking, drug inhibition, and metabolic transport in infected cells	Monitoring of lipid trafficking, drug inhibition, and metabolic transport in virus-infected cells; Visualization of lipid rafts during virus assembly
**Physicochemical actuators/biosensors**	pHluorin, pHoenix and SRpHi_1-4_ (pH), TrxRFP1 and Peredox (redox), GEVIs (voltage), NOA-1 (nitric oxide), OptoGEF-RhoA (contractile forces)	Modulation/monitoring of physicochemical parameters in pathogen or host cell organelles	Modulation/monitoring of physicochemical parameters in pathogen or host cell organelles	Modulation/monitoring of physicochemical parameters in host cell organelles

Selected abbreviations: CIBN, N-terminus of calcium and integrin-binding protein 1; cNMP, cyclic nucleotide monophosphate; GECA, genetically encoded calcium actuators; GECI, gene-encoded calcium indicator; GFP, green fluorescent protein; GPCR, G protein-coupled receptor; LACE, light-activated CRISPR-Cas9 effector; LAPD, light-activated phosphodiesterase; LITE, light-inducible transcriptional effectors; PACR, photoactivable Ca^2+^ releaser; PASS, phosphatidic acid biosensor with superior sensitivity; PIF, phytochrome interacting factor; PKD, protein kinase D; ROS, reactive oxygen species

## Light and seek: A eukaryote hiding within a eukaryote

Parasites include an assorted group of eukaryotic pathogens taking advantage of the host, which itself is also a eukaryotic organism. The study of the complex relationship between parasites and host cells is often compromised because chemical modulators usually cannot distinguish between targets conserved in both entities. Likewise, a spatiotemporal detection of universal metabolites in intracellularly residing parasites is simply not possible via chemical methods. Besides troubleshooting these issues, making of optogenetic strains allows us to literally tell the pathogen (or host cell) when and where to modulate or monitor the cascade and for how long and how much. A pioneering study involving expression of a light-activated adenylate cyclase in the protozoan parasite *Toxoplasma gondii* has already demonstrated the proof of principle for applying optogenetics in infection research [4]. Equally, expression of gene-encoded biosensors has enabled an otherwise challenging monitoring of subcellular calcium in *T*. *gondii* and *Plasmodium falciparum* enclosed within host cells [8,9]. These works have indeed paved a way to tap the vast potential of optogenetic actuators and biosensors. One can, for example, evaluate the roles of specific molecules during various parasitic stages, as epitomized by *T*. *gondii* and *Trypanosoma cruzi* (Fig 2A). Some of the most fascinating applications in parasites involve perturbation of ion homeostasis by light-gated channels, as well as photo-oscillation of calcium, cNMP (cyclic nucleotide monophosphate), and phosphoinositide signaling cascades. In essence, a rigorous experimental design can permit systematic dissection of signaling by studying optically induced changes in histone coding, transcriptome, proteome, and metabolome.

A repertoire of cation- and anion-specific channelrhodopsin variants is available to investigate inter- or intra-organelle ion homeostasis [10]. Evenly, genetically encoded calcium actuators (GECAs) [11,12] and light-induced cyclic nucleotide cyclases [13–16], along with corresponding phosphodiesterases [17,18], are perfectly poised to elucidate novel aspects of calcium and cNMP signaling, respectively. For instance, the parasite strains expressing a light-activated adenylate or guanylate cyclase in a PKA- (cAMP-dependent protein kinase) or PKG-deficient (cGMP-dependent protein kinase) mutant can be subjected to phosphoproteomic analysis to identify the core signaling mediators. On a different note, the method may even prove beneficial over ablation of native signaling proteins in certain cases because a sophisticated reversible control can be achieved as opposed to all or none effect in the gene-knockout mutants. This is well exemplified by applying optogenetics in *T*. *gondii* [4], in which induction of parasite-derived cytosolic cAMP can exert contrary effects depending on the duration and intensity of the stimulus. In this regard, it does make sense to couple optical regulation with a real-time detection using apposite sensors. A gamut of color-tuned biosensors, such as for calcium [19–21], cAMP [22], cGMP [23], c-di-GMP [24], DAG (diacylglycerol) [25], and IP_3_ [26], is available to quantify subcellular oscillations.

Conversely, the above tools can be expressed in host cells to study the influence of host milieu on parasites. One such example is to dissect the mechanism of action of antitrypanocidal drugs, which control the muscle function by apparent modulation of calcium homeostasis during chronic Chagas disease [27]. These drugs are also active against *Leishmania* [28], further advocating the utility of calcium releasers and sensors (Table 1). Other appealing optogenetic approaches involve engineering host cells or parasites to harbor reactive oxygen species generating proteins (RGPs) [29], as well as the biosensors of lipids [25,30,31], polar metabolites [32–34], nitric oxide [35], voltage [36], redox [37,38], and pH [39–41], each of them tailored to address specific paradigms and individual needs (Table 1).

## Exposing bugs as well as the bunker: A prokaryote inside a eukaryote

Just as with intracellular parasites, infection with prokaryotic pathogens can be examined from the side of the bacteria as well as from the host-cell side. Yet again, studying pathogen–host interactions has so far not relied on the deployment of optogenetics. Table 1 lists some customary optogenetic tools, which are projected to elucidate the mechanisms underlying the uptake of bacteria, survival within host cells, or transcytosis for leading human pathogens including but not limited to *Mycobacteria*, *Chlamydia*, *Salmonella*, and *Staphylococcus*. As depicted in Fig 1A and Table 1, the standard tools when engineering prokaryotes may use proteins with a photo-active chromophore (LOV [light, oxygen, and voltage], FAD [flavin adenine dinucleotide], BLUF [blue light sensing using FAD]) that can activate downstream cascades by altering the secondary messengers, such as cAMP [13,14], cGMP [15,16], or c-di-GMP [42,43]. It is also possible to develop light-responsive expression systems using photocaged effectors, such as doxycycline [44] and IPTG (isopropyl β-D-1-thiogalactopyranoside) [45] or proteins [46,47], which are released or activated upon illumination to control the gene expression or protein activity. Just recently, the LOV2-ODC-degron system has been reported, which targets the conjugated protein of interest to light-dependent proteasomal degradation, thereby controlling the protein stability [48] (Fig 1B). Even though most of these tools have been originally developed in eukaryotic cells, their tailored adoption in prokaryotes is quite plausible. Other comparable applications include modulation of protein–protein interactions, which could in turn be used to regulate cell signaling, genome editing, endogenous transcription, and epigenetic states [49–53] (Fig 1B). Equally, gene-encoded sensors can be applied to monitor the pH, ions, membrane potential, redox states, temperature, pressure, and molecular crowding [54].

Similar approaches can be used to modify host cells and regulate gene expression, signaling, autophagy, and organelle functions—processes that are considered important for interactions of host cells with bacterial pathogens, e.g., *Chlamydia* (Fig 2A). Optogenetics also enables the control of organelle transport and positioning [55], which might be useful when studying the importance of host organelle hijacking by microbes. It is even possible to induce and repress the mechanotransduction and cellular forces with spatiotemporal accuracy [56]. The underlying method involves controlling the subcellular activation of RhoA using the CRY2/CIBN light-gated dimerizer system [50]. In order to apply these methods, some hurdles need to be overcome, especially when manipulating mammalian host cells. The first one is the delivery of optogenetic proteins, which can be best solved by making stable transgenic lines using the established viral expression systems. Another issue is the compartmentalization, i.e., the targeting of a tool to the chosen organelle, such as nucleus, endoplasmic reticulum, and mitochondria. It can be resolved, though, by introducing organelle-specific signal sequences into the protein of interest. Parallel measurement of metabolites and ions in host cells harboring a pathogen is another bottleneck, which can be solved by coexpression of suitable biosensors. Once inducted, an effective commissioning of these tools is expected to shed light on numerous processes that have remained shadowy for a while.

## Sunshine everywhere: When the light gets viral

Viruses are master modulators of signaling, immune response, and metabolism in the infected host [57–59]. In a way similar to eukaryotic and prokaryotic pathogens, most applications are also applicable to viruses (Table 1); although, optogenetic tools have to be targeted primarily to the host cell (Fig 2B). For example, opsin and GECA family proteins allow in-depth examination of the role of ions in promoting or demoting the viral lifecycle. One could envisage optogenetically eliciting a release of Ca^2+^ from the endoplasmic reticulum (naturally occurring via IP_3_R in HIV-1, HSV [herpes simplex virus], rotavirus), activation of PLC pathway (mediated via GPCRs in rotavirus), impairment of SERCA (sarco-endoplasmic reticulum calcium ATPase), and control of membrane permeability (via viroporin in HIV-1, HCV, influenza virus, coronavirus) through light-gated channels or pumps [60,61]. Phosphoinositide actuators [62] can be applied to control early steps of attachment or fusion to the host membrane in the case of Ebola virus, coronavirus, and HIV-1 [63–65]. Other potential usages include light-mediated regulation of immune response (e.g., activation of HIV-infected CD4 cells), killing of virus-infected host cells (e.g., IFN_Υ_ in CD8^+^ and NK cells), and photo-editing of viral genomes. Similarly, ROS-yielding proteins, physicochemical actuators, protein recruiters/oligomerizers, nucleotide cyclases, and lipid-derived mediators allow us to study other enigmatic aspects (Table 1).

As indicated above, viruses can also be used as vectors for delivering optogenetic tools into specific cell populations or tissues in mammalian cells. Addgene repository contains several lentiviral constructs for targeted delivery and integration into genomes. Additional popular models include adeno-associated virus [66] and rabies virus [67]. Not least, selected gene-encoded sensors that can facilitate optogenetic work in virology include GECIs (gene-encoded calcium indicators), cNMP biosensors, as well as fluorescent indicators for pH, lipids, and several other metabolites (Table 1). Among many, one assay would be to test the pH dependence of membrane fusion during the entry of influenza and stomatitis viruses, mediated by hemagglutinin and G glycoprotein, respectively [68,69]. Another conceivable application is to visualize lipid rafts as a predicted platform for the entry, assembly, and release of viral particles [70–72]. Finally, a fusion approach coutilizing optical actuators and sensors will certainly embolden existing toolboxes in virology.

## No room for darkness: Concluding remarks

A modulation and monitoring of pathogen-specific pathways without affecting the sheltering host cell is nearly impossible with chemical modulators and fluorophores. Contrariwise, selective manipulation of the infected host is equally challenging. Although not common yet, optogenetics offers an imperative toolbox to modify and monitor subcellular processes both in the host as well as in the pathogen. The deployment of optogenetics shall overcome most, if not all, difficulties inherent to classic approaches in infection research. Just as with neurosciences, this will hopefully also lead to improvement of old tools and discovery of customized solutions catering pathogens in imminent future. Hence, building a bridge between the fields of optogenetics and infection biology remains more important than ever.

## Supporting information

S1 Appendix Major optogenetic tools and underlying source. (PDF)

## Abbreviations

cGMP: cyclic guanosine monophosphate
CIBN: N-terminus of calcium and integrin-binding protein 1
cNMP: (cyclic nucleotide monophosphate)
GECA: genetically encoded calcium actuator
GECI: gene-encoded calcium indicator
GFP: green fluorescent protein
GPCR: G protein-coupled receptor
LACE: light-activated CRISPR-Cas9 effector
LAPD: light-activated phosphodiesterase
LITE: light-inducible transcriptional effector
PACR: photoactivable Ca^2+^ releaser
PASS: phosphatidic acid biosensor with superior sensitivity
PIF: phytochrome interacting factor
PKD: protein kinase D
ROS: reactive oxygen species
