# Supplementary material for: Illuminating pathogen–host intimacy through optogenetics
Source: PLoS Pathog. 2018 Jul 12;14(7):e1007046. doi: 10.1371/journal.ppat.1007046 (PMC6042787; doi:10.1371/journal.ppat.1007046)
Supplement: S1 Appendix — (PDF) [file ppat.1007046.s001.pdf]

Illuminating Pathogen-Host Intimacy through Optogenetics

Ruben D. Arroyo-Olarte, Laura Thurow, Vera Kozjak-Pavlovic and Nishith Gupta

S1 Appendix: Major optogenetic tools and underlying sources

Protein Family	Protein/Function	Addgene Constructs (selected)	References
Opsin	Channelrhodopsin<br>(K^+, Na^+, Ca^{2+}, Cl^-)	Channelrhodopsin: 15753, 20939, 20071, 98171, 66709	[1–3]
GECA, GECI	PACR (photoactivable Ca^{2+} releaser), Light-activated GPCRs/RTKs, OptoSTIM1, GCaMP$_{1-6}$, YC-Nano	PACR: 55774, 55775<br>GPCR(OptoXRs): 20947, 20948<br>RTK: not available *via* Addgene<br>OptoSTIM1: 70159, 70160<br>GCaMP6$_{f,m,s}$: 40753, 40754, 40755<br>YC-Nano: 51961, 51964, 51966	[4–10]
Cyclic nucleotide cyclase and sensor	bPAC and mPAC for cAMP, BeCyclOp (RhoGC) and bPGC (BlgC) for cGMP, BphS for c-di-GMP, Flamindo$_{1-3}$ (cAMP), cGi and FlincG$_{1-3}$ (cGMP), RNA-based sensor (c-di-GMP)	bPAC: 85468, 28134<br>mPAC: Not available *via* Addgene<br>BeCyclOp (RhoGC): 85469, 66779<br>Flamindo$_{1-3}$: 73938, 102356<br>FlincG$_{1-3}$: 49202, 49203, 49204<br>RNA-based sensor: 79158-61	[11–20]
Phosphodiesterase	LAPD (cAMP, cGMP), BlrP1 and EB1 (c-di-GMP)	Not Available *via* Addgene	[21–23]
Genome editing, transcription, protein stability and epigenetics	LACE, LOV2-ODC/B-LID degron, LITE	LACE: 60552-54, 60718<br>LOV2-ODC/B-LID degron: 49570<br>LITE: 47450-58, 48253	[24–27]
ROS generating proteins	KillerRed (superoxide), miniSOGs (singlet oxygen)	KillerRed: 45761, 74748<br>miniSOG: 54824, 54821, 54801	[28–30]
Lipid actuators	CRY2/CIBN fusion coupled to inositol phosphatase	66841, 79561, 79562, 79565, 79567	[31]
Protein recruiters and oligomerizers	(1) CRY2/CIBN fusion to Cre system<br>(2) CRY2/CIBN fusion to GTPase<br>(3) CRY2 fusion to antiviral oligomers<br>(4) PhyB/PIF coupled to Tiam-DH-PH	(1) 75367, 75368, 79574, 58368<br>(2) 42954, 42955, 42958, 42959<br>(3) Not available *via* Addgene<br>(4) 22276, 22277, 22279, 22474	[32–36]
Gene-encoded metabolite sensors	Lact-C2-GFP (PtdSer), PKCδ-C1 and PKD-C1 (diacylglycerol), PASS (PtdOH), small soluble metabolite sensors (sugars, amino acids, lactate *etc.*)	Lact-C2-GFP: 22852<br>PKCδ-C1 & PKD-C1: 21190, 21204, 21205<br>PASS: Not available *via* Addgene<br>Metabolite sensors: 66843, 66845	[37–42]
Physicochemical actuators/biosensors	pHoenix, pHluorin and SRpHi$_{1-4}$ (pH), TrxRFP1 and Peredox (redox), GEVIs (voltage), NOA-1 (nitric oxide), OptoGEF-RhoA (contractile forces)	pHoenix: 70111, 70112<br>pHluorin: 70113, 73794<br>SRpHi$_{1-4}$: 102541-44<br>TrxRFP1: 98996<br>Peredox: 32380<br>GEVIs: 101655, 101658<br>OptoGEF-RhoA: 42959	[43–50]

Selected Abbreviations: BeCyclOp, *Blastocladiella emersonii* guanylate cyclase opsin; B-LID, blue light inducible degradation; BlrP1, blue light regulated phosphodiesterase 1; bPAC, *Beggiatoa* photo-activated adenylate cyclase; CIBN, N-terminus of calcium and integrin-binding protein 1; CRY2, cryptochrome 2 (*Arabidopsis*); GECA, gene-encoded calcium actuator; GECI, gene-encoded calcium indicator; GEVI, gene-encoded voltage indicator; GPCR, G protein-coupled receptor; LACE, light-activated CRISPR-Cas9 effector; LAPD, light-activated phosphodiesterase; LITE, light-inducible transcriptional effector; LOV, light, oxygen and voltage;

miniSOG, mini singlet oxygen generator; mPAC, *Microcoleus* photo-activated adenylate cyclase; PACR, photoactivable Ca^{2+} releaser; PASS, phosphatidic acid biosensor with superior sensitivity; PIF, phytochrome interacting factor; PhyB, phytochrome B; ODC, ornithine decarboxylase; RGP, ROS-generating protein; ROS, reactive oxygen species; SRpHi, super-resolution pH indicator; YC-Nano, yellow cameleon Nano

References

1. Nagel G, Ollig D, Fuhrmann M, Kateriya S, Musti AM, Bamberg E, et al. Channelrhodopsin-1: a light-gated proton channel in green algae. Science. 2002; 296: 2395–2398
2. Nagel G, Szellas T, Huhn W, Kateriya S, Adeishvili N, Berthold P, et al. Channelrhodopsin-2, a directly light-gated cation-selective membrane channel. Proc Natl Acad Sci USA. 2003; 100: 13940–13945
3. Wietek J, Wiegert JS, Adeishvili N, Schneider F, Watanabe H, Tsunoda SP, et al. Conversion of channelrhodopsin into a light-gated chloride channel. Science. 2014; 344: 409–12
4. Fukuda N, Matsuda T, Nagai T. Optical control of the Ca^{2+} concentration in a live specimen with a genetically encoded Ca^{2+}-releasing molecular tool. ACS Chem Biol. 2014; 9: 1197–1203
5. Airan RD, Thompson KR, Fenno LE, Bernstein H, Deisseroth K. Temporally precise in vivo control of intracellular signalling. Nature. 2009; 458: 1025–1029
6. Kim N, Kim JM, Lee M, Kim CY, Chang KY, Heo W Do. Spatiotemporal control of fibroblast growth factor receptor signals by blue light. Chem Biol. 2014; 21: 903–912
7. Kyung T, Lee S, Kim JE, Cho T, Park H, Jeong Y-M, et al. Optogenetic control of endogenous Ca^{2+} channels in vivo. Nat Biotechnol. 2015; 33: 1092–1096
8. Miyawaki A, Llopis J, Heim R, McCaffery JM, Adams JA, Ikura M, et al. Fluorescent indicators for Ca^{2+} based on green fluorescent proteins and calmodulin. Nature. 1997; 388: 882–887
9. Chen T-W, Wardill TJ, Sun Y, Pulver SR, Renninger SL, Baohan A, et al. Ultrasensitive fluorescent proteins for imaging neuronal activity. Nature. 2013; 499: 295–300
10. Horikawa K, Yamada Y, Matsuda T, Kobayashi K, Hashimoto M, Matsu-Ura T, et al. Spontaneous network activity visualized by ultrasensitive Ca^{2+} indicators, yellow Cameleon-Nano. Nat Methods. 2010; 7: 729–732
11. Ryu M-H, Moskvin O V., Siltberg-Liberles J, Gomelsky M. Natural and engineered photoactivated nucleotidyl cyclases for optogenetic applications. J Biol Chem. 2010; 285: 41501–41508
12. Stierl M, Stumpf P, Udwari D, Gueta R, Hagedorn R, Losi A, et al. Light modulation of cellular cAMP by a small bacterial photoactivated adenylyl cyclase, bPAC, of the soil bacterium *Beggiatoa*. J Biol Chem. 2011; 286: 1181–1188
13. Raffelberg S, Wang L, Gao S, Losi A, Gärtner W, Nagel G. A LOV-domain-mediated blue-light-activated adenylate (adenylyl) cyclase from the cyanobacterium *Microcoleus chthonoplastes* PCC 7420. Biochem J. 2013; 455: 359–365
14. Avelar GM, Schumacher RI, Zaini PA, Leonard G, Richards TA, Gomes SL. A rhodopsin-guanylyl cyclase gene fusion functions in visual perception in a fungus. Curr Biol. 2014; 24: 1234–1240
15. Scheib U, Stehfest K, Gee CE, Körschen HG, Fudim R, Oertner TG, et al. The rhodopsin-guanylyl cyclase of the aquatic fungus *Blastocladiella emersonii* enables fast optical control of cGMP signaling. Sci Signal. 2015; 8: rs8
16. Ryu MH, Gomelsky M. Near-infrared light responsive synthetic c-di-GMP module for optogenetic applications. ACS Synth Biol. 2014; 3: 802–810
17. Odaka H, Arai S, Inoue T, Kitaguchi T. Genetically-encoded yellow fluorescent cAMP indicator with an expanded dynamic range for dual-color imaging. PLoS One. 2014; 9: e100252
18. Harada K, Ito M, Wang X, Tanaka M, Wongso D, Konno A, et al. Red fluorescent protein-based cAMP indicator applicable to optogenetics and in vivo imaging. Sci Rep. 2017; 7: 7351
19. Bhargava Y, Hampden-Smith K, Chachlaki K, Wood KC, Vernon J, Allerston CK, et al. Improved genetically-encoded, FlincG-type fluorescent biosensors for neural cGMP imaging. Front Mol Neurosci. 2013; 6: 26
20. Wang XC, Wilson SC, Hammond MC. Next-generation RNA-based fluorescent biosensors enable anaerobic detection of cyclic di-GMP. Nucleic Acids Res. 2016; 44: e139

21. Barends TRM, Hartmann E, Griese JJ, Beitlich T, Kirienko NV, et al. Structure and mechanism of a bacterial light-regulated cyclic nucleotide phosphodiesterase. Nature. 2009; 459: 1015–1018
22. Gasser C, Taiber S, Yeh C-M, Wittig CH, Hegemann P, Ryu S, et al. Engineering of a red-light-activated human cAMP/cGMP-specific phosphodiesterase. Proc Natl Acad Sci USA. 2014; 111: 8803–8808
23. Ryu MH, Fomicheva A, Moskvin O V, Gomelsky M. Optogenetic module for dichromatic control of c-di-GMP signaling. J Bacteriol. 2017; 199. e00014-17
24. Polstein LR, Gersbach CA. A light-inducible CRISPR-Cas9 system for control of endogenous gene activation. Nat Chem Biol. 2015; 11: 198–200
25. Renicke C, Schuster D, Usherenko S, Essen LO, Taxis C. A LOV2 domain-based optogenetic tool to control protein degradation and cellular function. Chem Biol. 2013; 20: 619–626
26. Bonger KM, Rakhit R, Payumo AY, Chen JK, Wandless TJ. General method for regulating protein stability with light. ACS Chem Biol. 2014; 9: 111–115
27. Konermann S, Brigham MD, Trevino A, Hsu PD, Heidenreich M, et al. Optical control of mammalian endogenous transcription and epigenetic states. Nature. 2013; 500: 472–476
28. Bulina ME, Chudakov DM, Britanova OV, Yanushevich YG, Staroverov DB, Chepurnykh TV, et al. A genetically encoded photosensitizer. Nat Biotechnol. 2006; 24: 95–99
29. Bulina ME1, Lukyanov KA, Britanova OV, Onichtchouk D, Lukyanov S, Chudakov DM. Chromophore-assisted light inactivation (CALI) using the phototoxic fluorescent protein KillerRed. Nat Protoc. 2006; 1: 947–953
30. Shu X, Lev-Ram V, Deerinck TJ, Qi Y, Ramko EB, Davidson MW, et al. A genetically encoded tag for correlated light and electron microscopy of intact cells, tissues, and organisms. McIntosh JR, editor. PLoS Biol. 2011; 9: e1001041
31. Idevall-Hagren O, Dickson EJ, Hille B, Toomre DK, De Camilli P. Optogenetic control of phosphoinositide metabolism. Proc Natl Acad Sci USA. 2012; 109: E2316-23
32. Kennedy MJ, Hughes RM, Peteya LA, Schwartz JW, Ehlers MD, Tucker CL. Rapid blue-light-mediated induction of protein interactions in living cells. Nat Methods. 2010; 7: 973–975
33. Taslimi A, Zoltowski B, Miranda JG, Pathak GP, Hughes RM, Tucker CL. Optimized second-generation CRY2-CIB dimerizers and photoactivatable Cre recombinase. Nat Chem Biol. 2016; 12: 425–30
34. Bugaj LJ, Choksi AT, Mesuda CK, Kane RS, Schaffer D V. Optogenetic protein clustering and signaling activation in mammalian cells. Nat Methods. 2013; 10: 249–252
35. Diner BA, Lum KK, Toettcher JE, Cristea IM. Viral DNA Sensors IFI16 and Cyclic GMP-AMP synthase possess distinct functions in regulating viral gene expression, immune defenses, and apoptotic responses during herpesvirus infection. mBio. 2016; 7: e01553-16
36. Levskaya A, Weiner OD, Lim WA, Voigt CA. Spatiotemporal control of cell signalling using a light-switchable protein interaction. Nature. 2009; 461: 997–1001
37. Yeung T, Gilbert GE, Shi J, Silvius J, Kapus A, Grinstein S. Membrane phosphatidylserine regulates surface charge and protein localization. Science. 2008; 319: 210–213
38. Oancea E, Teruel MN, Quest AFG, Meyer T. Green fluorescent protein (GFP)-tagged cysteine-rich domains from protein kinase C as fluorescent indicators for diacylglycerol signaling in living cells. J Cell Biol. 1998; 140: 485–498
39. Zhang F, Wang Z, Lu M, Yonekubo Y, Liang X, Zhang Y, et al. Temporal production of the signaling lipid phosphatidic acid by phospholipase D2 determines the output of extracellular signal-regulated kinase signaling in cancer cells. Mol Cell Biol. 2014; 34: 84–95
40. Paige JS, Nguyen-Duc T, Song W, Jaffrey SR. Fluorescence imaging of cellular metabolites with RNA. Science. 2012; 335: 1194
41. Rogers JK, Guzman CD, Taylor ND, Raman S, Anderson K, Church GM. Synthetic biosensors for precise gene control and real-time monitoring of metabolites. Nucleic Acids Res. 2015; 43: 7648–760
42. Rogers JK, Church GM. Genetically encoded sensors enable real-time observation of metabolite production. Proc Natl Acad Sci USA. 2016; 113:2388–2393
43. Rost BR, Schneider F, Grauel MK, Wozny C, G Bentz C, Blessing A, et al. Optogenetic acidification of synaptic vesicles and lysosomes. Nat Neurosci. 2015; 18: 1845–1852

44. Mahon MJ. pHluorin2: an enhanced, ratiometric, pH-sensitive green florescent protein. Adv Biosci Biotechnol. 2011; 2: 132–137
45. Richardson DS, Gregor C, Winter FR, Urban NT, Sahl SJ, Willig KI, et al. SRpHi ratiometric pH biosensors for super-resolution microscopy. Nat Commun. 2017; 8: 577
46. Fan Y, Makar M, Wang MX, Ai HW. Monitoring thioredoxin redox with a genetically encoded red fluorescent biosensor. Nat Chem Biol. 2017; 13: 1045–1052
47. Hung YP, Albeck JG, Tantama M, Yellen G. Imaging cytosolic NADH-NAD(+) redox state with a genetically encoded fluorescent biosensor. Cell Metab. 2011; 14:545–54
48. Xu Y, Zou P, Cohen AE. Voltage imaging with genetically encoded indicators. Curr Opin Chem Biol. 2017; 39: 1–10
49. Sato M, Hida N, Umezawa Y. Imaging the nanomolar range of nitric oxide with an amplifier-coupled fluorescent indicator in living cells. Proc Natl Acad Sci USA. 2005; 102: 14515-1420
50. Valon L, Marín-Llauradó A, Wyatt T, Charras G, Trepat X. Optogenetic control of cellular forces and mechanotransduction. Nat Commun. 2017; 8: 14396
